# Therapeutic Effects of Natural Products on Liver Cancer and Their Potential Mechanisms

**DOI:** 10.3390/nu16111642

**Published:** 2024-05-27

**Authors:** Jinhong Guo, Wenjie Yan, Hao Duan, Diandian Wang, Yaxi Zhou, Duo Feng, Yue Zheng, Shiqi Zhou, Gaigai Liu, Xia Qin

**Affiliations:** 1 Beijing Key Laboratory of Bioactive Substances and Functional Food, Beijing Union University, Beijing 100023, China; gjh121133@163.com (J.G.); meyanwenjie@buu.edu.cn (W.Y.); dhuanao@163.com (H.D.); spwdd2018@163.com (D.W.); 15239407080@163.com (Y.Z.); shiqizhougood@163.com (S.Z.); lgg010403@163.com (G.L.); 2 Institute of Food and Nutrition Development, Ministry of Agriculture and Rural Affairs, Beijing 100081, China; 15525926785@163.com; 3 College of Food Science and Engineering, Northwest A&F University, Yangling 712100, China; zheng_yue21@163.com; 4 Graduate Department, Beijing Union University, Beijing 100101, China

**Keywords:** natural products, liver cancer, therapeutic drugs, mechanisms of action

## Abstract

Liver cancer ranks third globally among causes of cancer-related deaths, posing a significant public health challenge. However, current treatments are inadequate, prompting a growing demand for novel, safe, and effective therapies. Natural products (NPs) have emerged as promising candidates in drug development due to their diverse biological activities, low toxicity, and minimal side effects. This paper begins by reviewing existing treatment methods and drugs for liver cancer. It then summarizes the therapeutic effects of NPs sourced from various origins on liver cancer. Finally, we analyze the potential mechanisms of NPs in treating liver cancer, including inhibition of angiogenesis, migration, and invasion; regulation of the cell cycle; induction of apoptosis, autophagy, pyroptosis, and ferroptosis; influence on tumor metabolism; immune regulation; regulation of intestinal function; and regulation of key signaling pathways. This systematic review aims to provide a comprehensive overview of NPs research in liver cancer treatment, offering a foundation for further development and application in pharmaceuticals and functional foods.

## 1. Introduction

Liver cancer is the third most common cause of cancer-related deaths worldwide, and is categorized into primary and secondary liver cancer [1]. 

Primary liver cancer encompasses hepatocellular carcinoma, cholangiocarcinoma, and mixed hepatocellular-cholangiocarcinoma, whereas secondary liver cancer, also known as hepatoblastoma, is comparatively less common. Projections suggest that by 2025, liver cancer incidence will surpass one million cases, with hepatocellular carcinoma (HCC) comprising 90% of diagnoses [2]. This alarming trend presents substantial economic and public health challenges. Consequently, there is a pressing demand to investigate more effective and cost-efficient treatment strategies.

Treatment options for liver cancer are varied, with surgical intervention remaining the primary approach. However, even after curative surgery, challenges such as cancer metastasis, high recurrence rates, and poor prognosis persist [3]. For patients with advanced HCC who are ineligible for transplantation or have failed local and regional therapies, chemotherapy drugs like sorafenib are often employed. However, their effectiveness is hindered by drug resistance and accompanied by numerous side effects [4,5]. Consequently, there is an urgent need to develop anticancer drugs that are more cost-effective, safer, and efficacious to address the limitations of current treatment modalities and medications.

Natural products (NPs) exhibit diverse biological activities, including anti-inflammatory and antioxidant properties, rendering them promising sources for developing novel therapies for liver cancer [6,7]. Extensive in vitro and in vivo studies have unveiled the anti-tumor potential of NPs against liver cancer. Examples of such NPs encompass natural polysaccharides, flavonoids, terpenes, alkaloids, polyphenols, quinones, and essential oils. These NPs hold the potential to ameliorate and combat liver cancer through multiple pathways.

This article commences with an overview of current treatment modalities and medications for liver cancer. Additionally, we conducted a comprehensive search across “Google Scholar”, “Web of Science”, and “X-Mol”, yielding 181 articles pertaining to liver cancer and natural compounds from 2018 to 2024. The search utilized keyword combinations such as “liver cancer AND natural compounds” and “hepatic cancer AND natural compounds”. Included studies met specific criteria: (1) animal in vivo experiments showcasing the anti-cancer effects of natural compounds on liver cancer; (2) cell-based in vitro experiments demonstrating the anti-cancer effects of natural compounds on liver cancer; (3) original research articles in English. Exclusions comprised retrospective studies and meta-analyses. Subsequently, the article evaluates the efficacy and mechanisms of action of natural products sourced from various origins against liver cancer. Its objective is to furnish a systematic synthesis of research on natural products for liver cancer treatment, furnishing a theoretical foundation for the development and application of such products in pharmaceuticals and functional foods.

## 2. Therapeutic Drugs and Methods of Liver Cancer Treatment

Liver cancer treatment has evolved into a multidisciplinary approach, typically customized to individual patients based on tumor staging, the intricate interaction with underlying liver disease, and the patient’s overall health status [8]. Various treatment modalities are available depending on the stage of the tumor. Early-stage liver cancer patients are often candidates for surgical resection, transplantation, or local ablation. Intermediate-stage liver cancer, characterized by multifocal tumors, may be managed with transarterial chemoembolization (TACE). Late-stage liver cancer diagnoses are more common, offering a broader array of treatment options, including chemotherapy, immunotherapy, targeted drug therapy, and various other interventions [9,10].

### 2.1. Surgical Treatment

Surgical intervention, comprising hepatic resection and liver transplantation, has historically been the cornerstone of curative treatment for liver cancer, offering the most favorable outcomes with a 5-year survival rate of around 70–80%. Typically, candidates eligible for resection must have tumors situated in surgically accessible locations, adequate hepatic reserve, a sufficient volume of remaining liver, and undergo evaluation based on clinical and biochemical indicators or liver blood volume determination [11]. 

Patients with cirrhosis and limited tumor burden may opt for liver transplantation, which offers 5-year and 10-year survival rates of 70% and 50%, respectively, with a 5-year recurrence rate of 10–15% [12,13]. Liver transplantation’s long-term outcomes are generally deemed superior to those of resection. However, the scarcity of liver donors poses a challenge, as patients awaiting transplantation also confront the risk of tumor progression.

### 2.2. Local (Ablative) Treatment

Local ablation therapy serves as a curative option for solitary liver tumors with a maximum diameter of ≤5 cm, as well as for multiple liver tumors with a total count of ≤3 and a maximum diameter of ≤3 cm. Ethanol injection, microwave ablation, radiofrequency ablation, and cryotherapy are among the common local ablation therapies employed for early to intermediate-stage liver cancer patients. Numerous studies have validated that the 5-year survival rate of early-stage small liver cancer treated with local ablation approaches is approximately 60%, exhibiting no statistical variance compared to surgical resection [14,15]. Hence, local ablation therapy is acknowledged as another treatment modality with the potential for localized cure of hepatocellular carcinoma (HCC), following surgical resection (inclusive of liver transplantation) [16]. 

### 2.3. Hepatic Artery Therapy

Transarterial chemoembolization (TACE) has gained widespread adoption globally as the standard treatment for intermediate-stage liver cancer patients [17,18]. TACE is capable of addressing a wider spectrum of tumors, ranging from individual small liver cancers that cannot be treated via ablation due to technical limitations to intermediate-sized nodular liver cancers. Presently, there are two primary types of TACE: conventional TACE (cTACE) and drug-eluting bead TACE (DEB-TACE). Radioembolization, also referred to as selective internal radiation therapy (SIRT), serves as an alternative treatment for intermediate-stage liver cancer patients who either do not respond to TACE or have contraindications. It effectively reduces large liver cancers beyond Milan criteria and can act as a bridge to transplantation [19]. 

### 2.4. Systemic Therapy-Chemotherapy

Systemic chemotherapy plays a pivotal role in liver cancer treatment, especially for patients ineligible for surgery, particularly those in advanced stages. Sorafenib and lenvatinib, both tyrosine kinase inhibitors, are commonly used as first-line treatment drugs for advanced liver cancer patients [20,21]. Second-line treatment options for advanced-stage liver cancer encompass regorafenib, cabozantinib, and ramucirumab [22,23,24]. However, due to the considerable resistance of hepatocellular carcinoma (HCC) and the potential hepatotoxicity of drugs in patients with underlying liver disease, there exists no current consensus on systemic chemotherapy regimens [25]. Medical oncologists need to consider factors such as liver reserve, treatment objectives, treatment availability, and eligibility for clinical trials when deliberating systemic chemotherapy. Moreover, patients with liver cancer frequently develop resistance to drug therapy, resulting in a poor prognosis and high rates of recurrence, with an overall recurrence rate as elevated as 70% [26].

### 2.5. Radiotherapy

Radiotherapy, commonly referred to as radiation therapy, is a local treatment method for tumors using radiation beams or radioactive isotopes, alongside surgery and chemotherapy, constituting the three main traditional cancer treatment methods. It offers a better option for treating liver cancer in intermediate- to advanced-stage patients either alone or in combination with other techniques, and for patients in the early stages who are not suitable for surgical resection. Currently, the primary modality of external radiotherapy is stereotactic body radiotherapy (SBRT) [27].

### 2.6. Immunotherapy

Immune therapy for liver cancer is an emerging treatment method that works by activating or enhancing the patient’s own immune system to attack cancer cells. It mainly includes immune checkpoint inhibitors, adoptive cell therapy, tumor vaccines, oncolytic viruses, and non-specific immunotherapy [28]. Presently, immune checkpoint inhibitors are at the forefront of immune therapy, targeting critical checkpoints like programmed cell death protein 1 (PD-1) and its ligand PD-L1, along with cytotoxic T-lymphocyte-associated antigen 4 (CTLA-4). Moreover, combining immune therapy with targeted drugs used in liver cancer treatment can elicit a synergistic effect [26].

### 2.7. TCM (Traditional Chinese Medicine) Therapy

**Figure 1:**
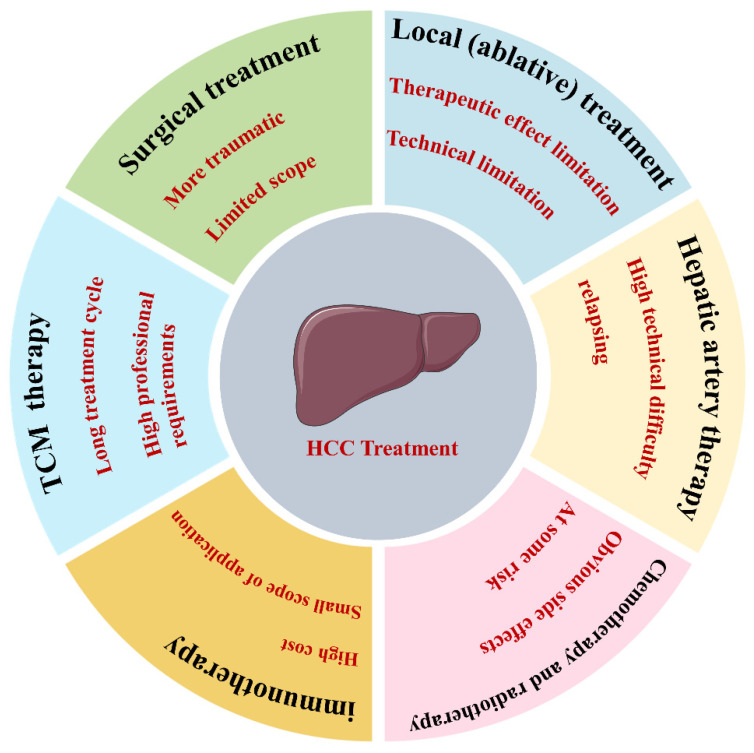
Current drugs and methods of treatment for liver cancer. Highlighting in red font indicates the limitations of the approach.

Currently, in clinical practice, anti-tumor drugs have shown limited efficacy in extending patient survival, and the outcomes of single-targeted therapy are also unsatisfactory. According to traditional Chinese medicine (TCM) theory, tumor diseases have diverse pathogenic factors, mainly characterized by deficiency syndrome with excess syndrome. TCM treatment emphasizes multi-targeting and holistic therapy, which can positively impact alleviating adverse reactions to chemotherapy, preventing tumor recurrence, and prolonging life [29]. In recent years, traditional Chinese medicine has demonstrated unique advantages in the prevention and treatment of primary liver cancer, significantly improving the quality of life, enhancing immunity, and prolonging life for liver cancer patients [30]. Figure 1 depicts the drugs and methods used to treat liver cancer and their limitations.

## 3. NPs with Therapeutic Activity for Liver Cancer

Based on the chemical structure and categories of compounds, we roughly classify NPs with therapeutic activity against liver cancer into seven specific categories: polysaccharides, flavonoids, terpenoids, alkaloids, polyphenols, quinones, and volatile oils. The therapeutic effects of each type of natural product on liver cancer, as well as their sources, doses, and mechanisms of action, will be detailed in the following sections.

### 3.1. Polysaccharides

Polysaccharides are biologically active macromolecules composed of 10 or more monomers linked by glycosidic bonds. Extensive research has highlighted the therapeutic and ameliorative effects of natural polysaccharides on liver cancer, sourced from plants, animals, marine organisms, and fungi [7]. For instance, polysaccharides purified from Panax ginseng (PHP-1) and active polysaccharides from Ganoderma lucidum (GLPS) function as tumor immunotherapy modulators. Both are capable of polarizing M2 macrophages into the M1 type by activating NF-κB and MAPK signaling pathways, thereby exerting anti-tumor activity [31,32].

Furthermore, high molecular weight polysaccharides extracted from Cordyceps sinensis not only enhance the immune function of mice but also induce tumor cell apoptosis through the Cytochrome-c/Caspase8/3 and IL-10/STAT3/Bcl2 pathways, thereby exerting anti-tumor effects [33]. In H22 hepatocellular carcinoma tumor-bearing mouse models, Ulva lactuca polysaccharide (UIP) extracted from green algae inhibits tumor growth by modulating gut microbiota and metabolite composition. Additionally, UIP has been found to suppress tumors in HepG2 and H22 cells by promoting ROS production [34]. Researchers, utilizing metabolomics, have demonstrated that Black fungus polysaccharide (BFP) inhibits tumor cell proliferation in H22 tumor-bearing mice by promoting DNA damage, weakening DNA damage repair, and inhibiting DNA synthesis [35].

Based on a large number of studies on the improvement of liver cancer by natural polysaccharides, natural polysaccharides have shown very good effects on immune regulation. On one hand, polysaccharides directly inhibit tumor growth through pathways such as blocking the cell cycle, inducing cell apoptosis, and inhibiting angiogenesis. On the other hand, polysaccharides can also regulate the host immune system, indirectly exerting anti-tumor effects by stimulating non-specific and specific immune responses [36]. Through the aforementioned review, it is evident that natural polysaccharides primarily exert their beneficial effects on liver cancer by inducing cell apoptosis, inducing cell cycle arrest, inhibiting angiogenesis, suppressing cancer cell invasion and metastasis, and modulating immune responses. In Table 1, we have compiled findings from 18 recently published studies elucidating the therapeutic effects of natural polysaccharides on liver cancer and their potential mechanisms of action.

### 3.2. Flavonoids

Flavonoid compounds are commonly found in various dietary components, including vegetables, fruits, wine, flowers, tea, and other plant-based food sources. They are celebrated for their myriad health benefits, encompassing anti-cancer, cardio-protective, anti-inflammatory, antimicrobial, antioxidant, and hepatoprotective properties. Extensive research underscores the therapeutic and preventive potential of plant-derived flavonoids against liver cancer. For instance, flavonoids isolated from Resina Draconis inhibit the progression of liver cancer by upregulating p21 expression driven by DNA damage, inducing cell apoptosis, and causing cell cycle G2/M phase arrest in human liver cancer cells [50]. Neobavaisoflavone (NBIF), a natural active ingredient derived from psoralen, exhibits anti-inflammatory, anti-cancer, and antioxidant properties. Researchers have found that NBIF induces mitochondrial outer membrane protein Tom20 alteration through ROS both in vivo and in vitro, thereby enhancing Bax recruitment to mitochondria and activating the calpain I-GSDME pathway in liver cancer cells to induce pyroptosis. Sinensetin (SIN), a polymethoxylated flavone found in citrus fruits, inhibits angiogenesis in liver cancer by targeting the VEGF/VEGFR2/AKT signaling pathway in a murine xenograft tumor model [51]. Studies indicate that extramedullary hematopoiesis (EMH) is a crucial mechanism for myeloid-derived suppressor cells (MDSCs) accumulation in tumor tissues, contributing to disease progression. Icaritin dampens tumoral immunosuppression, eliciting anti-tumor immune responses by preventing MDSC generation via EMH attenuation [52]. 

In summary, flavonoid compounds inhibit the progression of liver cancer by inducing cell apoptosis, blocking the cell cycle, inhibiting angiogenesis, and exerting antioxidant effects, consistent with previous research findings [53]. Additionally, flavonoid compounds also exhibit anti-cancer efficacy by inducing cell pyroptosis and modulating immune responses. This article summarizes 18 flavonoid compounds with therapeutic effects on liver cancer from the recent literature. Details regarding their sources, doses, and mechanisms of action are provided in Table 2.

### 3.3. Terpenoids

Terpenoids, a class of naturally occurring hydrocarbon compounds, are abundantly present in plants, especially coniferous trees. They are classified into various types based on the number of isoprene units in their structure, including monoterpenes, sesquiterpenes, diterpenes, triterpenes, tetraterpenes, and polyterpenes. Many terpenoids showcase notable biological activities, encompassing anti-inflammatory, anticancer, antimicrobial, and antiviral properties. As a result, they serve as invaluable resources for investigating natural products and advancing drug development initiatives [69]. A recent review has succinctly outlined the anticancer attributes of terpenoids, emphasizing their potent efficacy against tumors. This underscores their potential as additional avenues for the development of anticancer medications [70,71,72]. For liver cancer, some terpenoids have also demonstrated promising therapeutic effects.

Ginsenosides, vital active constituents of ginseng, exhibit a spectrum of therapeutic effects, including antioxidant, anti-inflammatory, vasodilatory, anti-allergic, and anticancer properties [73]. Recent research has elucidated the therapeutic mechanisms through which ginsenosides combat liver cancer. For instance, ginsenoside Rh4 impedes the progression of inflammation-related hepatocellular carcinoma by targeting the HDAC4/IL-6/STAT3 signaling pathway [74]. In a DEN-induced SD rat model, ginsenoside CK may suppress liver cancer cell proliferation by downregulating Bclaf1 expression, thereby inhibiting the HIF-1α-mediated glycolytic pathway and proliferation [75]. Both in vitro and in vivo studies demonstrate that ginsenoside Rk3 not only curtails cell proliferation and induces cell cycle arrest but also fosters HCC cell autophagy and apoptosis via the PI3K/AKT pathway [76]. Ginsenoside Rh2 and its octyl ester derivative (Rh2-O) suppress the invasion and metastasis of liver cancer through the c-Jun/COX2/PGE2 pathway [77]. Rh2-O also exhibits immune-regulatory effects on splenic lymphocytes in H22 tumor-bearing mice [78]. Betulinic acid inhibits the proliferation of liver cancer HUH7 and HCCLM3 cells by activating ferritinophagy in cancer cells, thereby inducing ferroptosis through the NCOA4/FTH1/LC3II signaling pathway [79]. Additionally, ganoderma triterpenoids regulate the gut microbiota to inhibit the progression of liver cancer in S180 and H22 tumor-bearing mice [80].

Therefore, terpenoids demonstrate anticancer effects through various mechanisms, including apoptosis induction, autophagy stimulation, ferroptosis initiation, cell cycle arrest, inhibition of cell migration and invasion, modulation of immunity, metabolism, and gut microbiota. Table 3 provides a summary of the therapeutic effects and potential mechanisms of terpenoids on liver cancer.

### 3.4. Alkaloids

Alkaloids are organic compounds containing nitrogen, derived from amino acids and found in plants, animals, and microorganisms. They are classified based on their chemical structure into various types, including pyridine alkaloids, isoquinoline alkaloids, indole alkaloids, terpenoid alkaloids, steroidal alkaloids, quinoline alkaloids, and others. Alkaloids of natural origin exhibit diverse pharmacological activities such as anti-inflammatory, immunomodulatory, and anticancer properties. Numerous studies have highlighted the therapeutic effects of natural alkaloids from various sources on HCC [89]. Matrine inhibits HCC invasion and migration by PTEN/Akt-dependent suppression of epithelial-mesenchymal transition (EMT) [90]. Anisodamine (ANI), an alkaloid extracted from Anisodus, exhibits effective therapeutic activity against HCC in xenograft mouse models. At a dose of 200 mg/kg/day, ANI inhibits the growth of HCC cells by suppressing the activation of the NLRP3 inflammasome, inducing cell apoptosis, and modulating levels of inflammatory factors [91]. Both in vitro and in vivo studies have shown the anti-HCC effects of epipolythiodioxopiperazine (ETP) alkaloids such as Chaetocochin J, primarily attributed to the inhibition of the PI3K/Akt/mTOR/p70S6K/4EBP1 pathway and downregulation of HIF-1α expression under hypoxic conditions, disrupting the binding of HIF-1α/p300. In a patient-derived xenograft model, Stachydrine hydrochloride (SH) regulates autophagy and cell cycle arrest via the LIF/AMPK axis, inducing cell senescence, thereby suppressing tumor initiation and progression in HCC [92]. Additionally, Cepharanthine extracted from Stephania cepharantha Hayata inhibits HCC cell growth and proliferation by modulating amino acid metabolism and suppressing tumor formation in vivo [93].

A recent review highlights the significant therapeutic potential of natural alkaloids for HCC [89]. In summary, the potential mechanisms of natural alkaloids in treating liver cancer are closely associated with inhibiting cell proliferation, migration, and invasion, blocking the cell cycle, inducing apoptosis and autophagy, regulating metabolism, and modulating immune function. Table 4 summarizes the therapeutic effects and potential mechanisms of alkaloids on liver cancer.

### 3.5. Polyphenol Compounds

Polyphenols, typically abundant in vegetables and fruits, serve as secondary metabolites in many plants and represent the richest natural source of antioxidants in the human diet. Numerous studies suggest that consuming polyphenol-rich foods can help prevent and treat common chronic diseases [104]. Due to their potent antioxidant and anti-inflammatory properties, polyphenols exhibit a significant therapeutic effect on liver cancer [105]. The fruits of Terminalia bellirica possess various pharmacological activities. Researchers purified crude extracts of TB to produce total tannin fractions (TB-TF) and found that TB-TF inhibited tumor growth in H22 tumor-bearing mice by inducing apoptosis, reducing angiogenesis, and modulating immune suppression in the tumor microenvironment [106]. Catechin acts on Hep-G2 and Huh-7 cells, inhibiting the proliferation of human liver cancer cells by suppressing the NF-κB signaling pathway and triggering mitochondrial apoptosis [107]. In vitro experiments demonstrate that curcumin inhibits the proliferation of liver cancer cells by reducing DJ-1 expression and inhibiting the PTEN/PI3K/AKT signaling pathway [108].In vivo studies suggest that curcumin improves diethylnitrosamine-induced liver cancer by regulating oxidative stress, inflammation, and gut microbiota [109]. Resveratrol induces apoptosis and inhibits proliferation, migration, and invasion of liver cancer cell lines (HepG2 and Hep3B), and suppresses HCC progression by downregulating MARCH1 expression [110].

In summary, polyphenolic compounds primarily inhibit the progression of liver cancer by inducing cell apoptosis, suppressing migration and invasion, regulating immunity, and improving gut microbiota abundance. Table 5 summarizes the effects and potential mechanisms of polyphenolic compounds in treating liver cancer.

### 3.6. Quinone 

Quinone compounds constitute a class of secondary metabolites in plants. Based on the number of benzene rings, quinone compounds can be divided into anthraquinones, naphthoquinones, benzoquinones, and phenanthraquinones [113]. Several studies have demonstrated the therapeutic activity of quinones against liver cancer. Upon acting on HepG2 cells, emodin induces autophagy and suppresses epithelial–mesenchymal transition (EMT) by inhibiting the PI3K/AKT/mTOR and Wnt/β-catenin pathways [114]. Additionally, a separate study found that a structural derivative of emodin, emodin succinyl ester (ESE), inhibits malignant proliferation and migration of hepatocellular carcinoma by suppressing the interaction between androgen receptor (AR) and enhancer of zeste homolog 2 (EZH2) [115]. Thymoquinone (TQ), the main bioactive component of black seed, has been shown to suppress tumor angiogenesis by regulating miR-1-3p [116]. Furthermore, plumbagin has been found to induce apoptosis by down-regulating GPX4 in a subcutaneous xenograft tumor model [117].

In summary, quinone compounds exert anticancer effects against liver cancer through the induction of apoptosis, autophagy, inhibition of migration and invasion, and suppression of angiogenesis. However, research on the role of quinone compounds in liver cancer is relatively limited, leaving room for exploration of their anti-tumor mechanisms and targets. Researchers can employ bioinformatics, computer simulation techniques, and molecular docking to analyze the molecular mechanisms and targets of quinone compounds in anti-tumor effects, providing a theoretical basis for subsequent experimental studies. Table 6 summarizes the anticancer effects and potential mechanisms of quinone compounds against liver cancer.

### 3.7. Volatile Oils 

Volatile oils are oily liquids found within plants, and research has highlighted their anti-tumor effects. In vitro and in vivo studies indicate that essential oil extracted from Artemisia argyi downregulates the mRNA and protein expression of DEPDC1, weakens Wnt/β-catenin signaling by reducing the production of Wnt1 and β-catenin, and prevents epithelial–mesenchymal transition (EMT) by downregulating vimentin and upregulating E-cadherin [118]. Additionally, essential oils extracted from the leaves of *Conobea scoparioides* Benth, Duguetia pycnastera Sandwith, and the bark of Aniba parviflora Mez inhibit the proliferation of HepG2 cells and suppress the growth of xenograft tumors in mice [119,120,121]. Pogostemon cablin essential oils, essential oil of lemon myrtle, and *Cyperus articulatus* L. rhizome essential oil exert anti-tumor effects by blocking the cell cycle and inducing apoptosis in liver cancer cells [122,123]. Table 7 summarizes the effects and potential mechanisms of volatile oils in treating liver cancer.

## 4. The Main Action Pathway and Potential Mechanism of Natural Compounds in the Treatment of Liver Cancer

**Figure 2:**
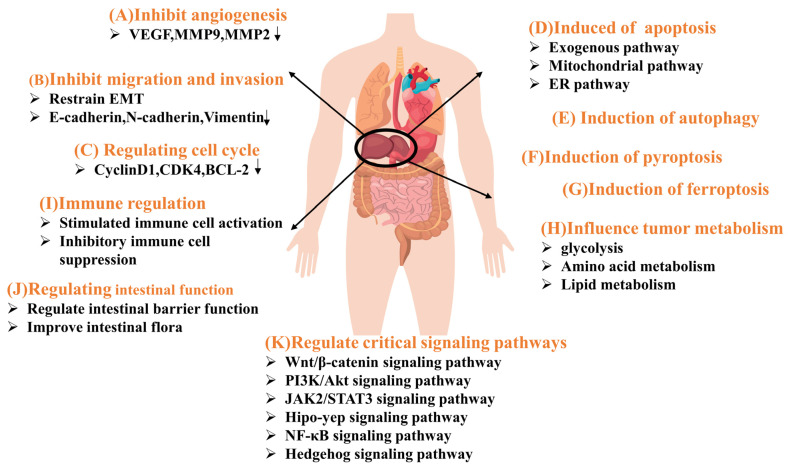
Mechanisms of NPs in treating liver cancer. A, B, C, D, E, F, G, H, I, J represent the primary pathways through which NPs treat liver cancer; K denotes the key signaling pathway through which NPs improve liver cancer.

**Figure 3:**
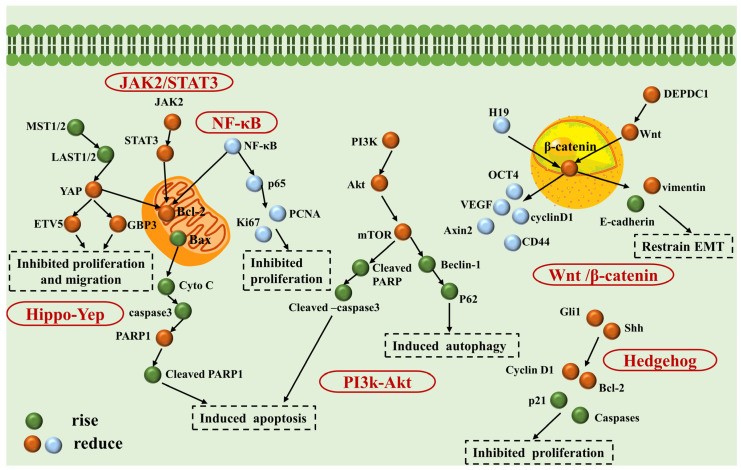
Mechanistic map of signaling pathways associated with NPs for liver cancer treatment. H19, long non-coding RNA H19; EMT, epithelial–mesenchymal transition.

The mechanism of action of NPs for treating liver cancer is currently under exploration in numerous studies. Here, we will detail the main pathways of action of natural compounds for liver cancer treatment. Utilizing multiple types of liver cancer cell lines in vitro and various animal models in vivo, studies have shown that the ameliorative and therapeutic effects of NPs on liver cancer primarily occur through eleven pathways. Figure 2 illustrates the mechanism of action of NPs in liver cancer treatment, while Figure 3 shows the diagram of signaling pathways associated with NPs treatment of liver cancer.

### 4.1. Inhibition of Angiogenesis

Cancer is characterized by highly vascularized solid tumors, where the continuous growth of new blood vessels supplies oxygen and nutrients to tumor cells, consequently fueling cancer progression [125]. Hence, anti-angiogenesis emerges as a promising approach for treating aggressively prognosed cancers [126,127].

The terpenoid compound saikosaponin b2 (SSb2), traditionally used in Chinese medicine for fever reduction and liver protection, has demonstrated anti-angiogenic effects both in vivo and in vitro. Its mechanism involves the inhibition of the VEGF/ERK/HIF-1α signaling pathway [82]. Thymoquinone (TQ), a quinone compound, intervened in DEN-induced hepatocellular carcinoma (HCC) in rats. The results revealed decreased expression of MMP2, MMP9, and VEGF in the rat liver, accompanied by increased levels of TIMP3 and miR-1-3p expression, suggesting that the anti-angiogenic effect of TQ in HCC is mediated through miR-1-3p regulation [116]. Natural polysaccharides like Asparagus polysaccharide, flavonoids like sinensetin, and polyphenols like tannins have also been found to exert anti-hepatocellular carcinoma effects by inhibiting tumor angiogenesis [51,106,128].

### 4.2. Inhibition of Migration and Invasion

Malignant cell invasion and migration significantly contribute to mortality in liver cancer patients. The process of tumor migration and invasion is intricate, involving multiple steps and factors, and regulated by various signaling pathways. Therefore, targeting these signaling molecules to disrupt tumor metastasis emerges as one of the strategies for treating liver cancer using natural compounds.

Epithelial–mesenchymal transition (EMT) stands as a pivotal process driving cancer cell migration. EMT is characterized by decreased cell adhesion and apical–basal polarity, ultimately fostering cell movement and invasion. Research findings suggest that ginsenoside Rh2 and its octyl ester derivative inhibit the invasion and metastasis of hepatocellular carcinoma via the c-Jun/COX2/PGE2 pathway [77]. Both in vivo and in vitro studies indicate that ginsenoside CK inhibits hypoxia-induced epithelial–mesenchymal transition in liver cancer cells through the HIF-1α/NF-κB feedback pathway [81]. The alkaloid CVB-D suppresses proliferation, migration, invasion, and EMT of HCC cell lines by inhibiting the EGFR-FAK-AKT/ERK1/2-Slug signaling pathway in human HCC [99]. Both in vivo and in vitro studies demonstrate that the alkaloid ventilagolin downregulates the expression of Pim-1, *N*-cadherin, and vimentin, upregulates the expression of E-cadherin, and inhibits the migration, invasion, and EMT of HCC cells [103]. These studies collectively confirm that NPs can exert anti-tumor effects by inhibiting migration and invasion.

### 4.3. Regulating Cell Cycle

The cell cycle is primarily regulated by cell cycle proteins, protein kinases, and kinase inhibitors. Dysregulation of these components can lead to aberrant cell cycle regulation, resulting in abnormal cell proliferation. Recent studies highlight that targeting the cell cycle is a key strategy in combating liver cancer [129]. Scutellaria barbata polysaccharides (SBP) disrupt the cell cycle at the G0/G1 phase by downregulating the expression levels of CyclinD1 and CDK4 in H22 tumor cells. Additionally, they upregulate the expression levels of p53 and Bax/Bcl-2, thereby inducing apoptosis in H22 tumor cells [130]. In vivo and in vitro studies have demonstrated that the flavonoid compound wogonin induces the degradation of cell cycle protein D1 by activating glycogen synthase kinase-3 beta (GSK3β), effectively blocking the cell cycle in H22 tumor cells [59]. Researchers have also discovered that the triterpenoid compound Cucurbitacin B (CuB) effectively impedes HCC progression by inducing DNA damage-dependent cell cycle arrest. This effect is mediated through the ataxia telangiectasia mutated (ATM)-dependent p53-p21-CDK1 and checkpoint kinase 1 (CHK1)-CDC25C signaling pathways [131]. Additionally, the alkaloid Stachydrine hydrochloride, volatile oils extracted from plants, and the polyphenolic compound Proanthocyanidin-B2 all exert anti-cancer effects by regulating the cell cycle [92,111,123].

### 4.4. Induction of Apoptosis

Apoptosis, also known as programmed cell death, is a self-regulatory process that cells undergo during development and aging to maintain dynamic equilibrium [132]. Three different pathways can initiate apoptosis: the extrinsic or cell surface death receptor pathway, the mitochondrial pathway, and the endoplasmic reticulum (ER) pathway [133]. Cancer cells tend to develop the ability to evade apoptosis, thereby resisting the effects of drug therapy [134]. Therefore, targeting apoptosis-related signals to induce cancer cell death is a critical mechanism of action for natural product anticancer activity. The aforementioned seven natural compounds all exert their anti-liver cancer effects by inducing apoptosis. Researchers have discovered that water-soluble polysaccharides extracted from Eucommia ulmoides leaves induce apoptosis via the mitochondrial pathway in H22 tumor-bearing mice [47]. The flavonoid compound lysionotin, upon acting on liver cancer cells and xenograft mouse models, enhances the expression levels of PARP, FasL, Bax, Bad, and cleaved caspases, while reducing the expression level of Bcl-xL. This leads to the induction of apoptosis through the caspase-3 mediated mitochondrial pathway [57]. The diterpenoid compound pseudolaric acid B triggers apoptosis in hepatocellular carcinoma cells by activating the AMPK/JNK/DRP1/mitochondrial fission pathway [86]. Additionally, the alkaloid protopine inhibits the viability of liver cancer cells, induces caspase-dependent apoptosis via an intrinsic pathway, and induces ROS, further blocking the PI3K/Akt signaling pathway [135].

### 4.5. Induction of Autophagy

Autophagy refers to the process, mediated by autophagy-related genes, of using lysosomes to degrade damaged organelles and certain macromolecules, playing a unique role in maintaining cellular homeostasis, and is considered a form of programmed cell death [136]. Aberrations in autophagy can lead to various pathological conditions, including cancer. While autophagy is generally seen as a survival mechanism, excessive activation can result in non-apoptotic cell death. Therefore, regulating autophagy presents an opportunity to improve the cure rate of liver cancer, offering new treatment targets and directions [137]. Studies have shown that the alkaloid veratramine significantly inhibits the proliferation, migration, and invasion of HepG2 cells and induces autophagy-mediated cell death by inhibiting the PI3K/Akt/mTOR signaling pathway [96]. Both in vitro and in vivo studies have demonstrated that the main active component, total flavonoids of Hedyotis diffusa Willd, induces endoplasmic reticulum stress (ER stress) and activates the PERK/EIF2α/ATF4 signaling pathway, inducing apoptosis and activating autophagy in HCC cells [60].

### 4.6. Induction of Pyroptosis

Pyroptosis, also known as inflammatory necrosis of cells, is a novel form of programmed cell death characterized by cell swelling and the formation of pores on the plasma membrane surface [138]. Gasdermin D (GSDMD) and gasdermin E (GSDME), members of the gasdermin family, serve as hallmarks for inducing pyroptosis through proteolytic fragmentation catalyzed by caspases 1/4/5/11 and caspase 3, respectively [138,139]. Research indicates that the activation of pyroptosis can promote the death of liver cancer cells, thereby exhibiting its anti-cancer properties [139]. Consequently, promoting pyroptosis in liver cancer cells may serve as a promising new therapeutic target for treating liver cancer. In vitro and in vivo studies have shown that flavonoid compound Neobavaisoflavone (NBIF) promotes ROS production in HCC cells, affecting Tom20 protein expression. This facilitates Bax recruitment to mitochondria, activating caspase-3, cleaving GSDME, and inducing pyroptosis [54]. The terpenoid compound Mallotucin D promotes cytochrome C release from mitochondria into the cytoplasm, leading to caspase-9 and caspase-3 cleavage, inducing GSDMD-related pyroptosis [87]. The alkaloid ajmalicine activates ROS-induced pyroptosis, exerting anti-tumor effects through the non-canonical caspase-3-GSDME pyroptosis pathway [140].

### 4.7. Induction of Ferroptosis

Iron death is a novel regulatory mechanism of cell demise, distinct from previously known forms like necrosis, apoptosis, and autophagy. It involves disruptions in cellular iron metabolism and accumulation of peroxides. Iron death plays a crucial role in preventing and treating HCC. Activating ferroptosis in liver cancer cells and enhancing their sensitivity to ferroptosis can induce cell death, thereby preserving normal liver function [141,142].

Hepcidin, a key regulator of iron metabolism, is expressed at higher levels in HCC tumor tissues. Dandelion polysaccharide (DP) regulates iron overload in HCC cells and transplant tumors by inhibiting interleukin-6 (IL-6)-induced JAK-STAT signaling, exerting anti-tumor effects [46]. The terpenoid compound heteronemin induces intracellular ROS formation and inhibits cell apoptosis via the p38/JNK MAPK signaling pathway. Additionally, it suppresses glutathione peroxidase 4 (GPX4) expression, a critical regulator of lipid peroxidation and iron metabolism, leading to lipid peroxide accumulation and iron death, thereby inhibiting HCC progression [88]. 

### 4.8. Influence Tumor Metabolism

Metabolic abnormalities play a crucial role in tumor survival and progression [143]. Abnormal tumor metabolism can affect glycolysis [144], amino acid metabolism [145], and lipid metabolism [146]. Targeting metabolic dysregulation within the organism can prevent and combat the development of liver cancer, which holds significant implications for identifying novel cancer management and treatment strategies.

Daidzin (DDZ), a natural brassin-like compound derived from soybeans, has been shown in in vitro and in vivo studies to interfere with the survival and migration of hepatocellular carcinoma (HCC) cells by downregulating the expression of TPI1, a gene involved in the glycolysis pathway and prognostically relevant for HCC [67].

Cepharanthine (CEP), an alkaloid extracted from Stephania cepharantha Hayata, has shown efficacy in inhibiting the proliferation of HCC cells by modulating cellular metabolism, particularly amino acid and cholesterol metabolism [93]. Moreover, through LC-MS metabolomics and lipidomics analyses, researchers have identified that the active components of cepharanthine injection impede the progression of HepG2 cells by disrupting lipid, energy, and amino acid metabolism [147].

### 4.9. Immune Regulation

In recent years, immunotherapy has gained traction for improving liver cancer treatment by countering the liver’s immune-suppressive environment, which enables cancer cells to evade therapy. NPs offer promising immunomodulatory effects, revitalizing immune surveillance and bolstering responses against liver cancer. They achieve this by targeting multiple immune pathways, either by boosting immune activators or by inhibiting suppressive factors, thus exerting anti-tumor effects [137].

#### 4.9.1. Activation of Stimulatory Immune Cells

The tumor microenvironment (TME) is pivotal in driving tumor initiation and progression, comprising a complex ecosystem of tumor cells, immune cells, and other surrounding cells [148]. In HCC, immune cells within the TME can be broadly categorized into immunostimulatory lymphoid cells and immunosuppressive myeloid cells and lymphocytes based on their differentiation and function.

Representative stimulatory immune cells include cytotoxic CD8+ T cells, CD4+ T cells with a type 1 helper phenotype, and natural killer (NK) cells. These cells play crucial roles in anti-tumor immunity [137].

Activation of stimulatory immune cells to promote the release of anti-tumor pro-inflammatory cytokines is one of the mechanisms through which natural products inhibit tumor growth in HCC immunotherapy. One study found that the polysaccharide from wild Cordyceps significantly inhibited tumor growth and metastasis in H22 tumor-bearing mice, improved blood routine indicators, increased the thymus index, decreased the spleen index, and increased the proportion of CD4+ T cells, CD8+ T cells, and macrophages. It exhibited significant anti-tumor activity by enhancing host immune function [33]. Furthermore, a polysaccharide (ESPS) purified from Eupolyphaga sinensis Walker promoted lymphocyte proliferation and inhibited liver cancer cell growth by enhancing lymphocyte activity in vitro, primarily natural killer (NK) cells. Additionally, ESPS markedly enhanced immunity in H22-bearing mice by increasing the spleen and thymus indices and effectively inhibiting H22 cell growth in vivo [149]. Safflower yellow (SY) is the primary active ingredient isolated from the traditional Chinese medicine Carthamus tinctorius. Studies have shown that SY degrades collagens via the TGF-β/Smad signaling pathway to promote infiltration of CD8+ T cells and Gr-1+ macrophages. Additionally, it can regulate the gut microbiota to enhance hepatic immune infiltration, thereby inhibiting the development of HCC [66].

#### 4.9.2. Suppression of Inhibitory Immune Cells

In addition to stimulatory immune cells, the TME also hosts inhibitory immune cells, notably regulatory T (Treg) cells, myeloid-derived suppressor cells (MDSCs), and tumor-associated macrophages (TAMs). These cells secrete diverse pro-inflammatory cytokines, which not only fuel cancer cell proliferation but also dampen the activity of stimulatory immune cells. In specific scenarios, natural products (NPs) demonstrate anti-tumor efficacy by thwarting the activation of inhibitory immune cells and curtailing the release of pro-tumorigenic cytokines [137].

Treg cells play a significant role as inhibitory immune cells in the HCC microenvironment. Studies have shown that resveratrol can inhibit tumor growth in HCC mouse models by reducing the number of CD8+CD122+ Tregs, downregulating immunosuppressive cytokines TGF-β1 and interleukin-10, and upregulating anti-tumor cytokines TNF-α and IFN-γ, thus reversing the immunosuppressive tumor microenvironment [150].

Myeloid-derived suppressor cells (MDSCs) are a group of immature immunosuppressive cells that help establish an immune-suppressive microenvironment in hepatocellular carcinoma (HCC). Recent studies have demonstrated that splenic extramedullary hematopoiesis (EMH) is an important mechanism for the accumulation of MDSCs in tumor tissues, thus contributing to disease progression. Icaritin, a prenylflavonoid derivative from plants of the Epimedium genus, has been shown to suppress tumor-associated splenic EMH, thereby inhibiting the production and activation of MDSCs and increasing the number and activity of cytotoxic T cells. As a result, it suppressed tumor progression and significantly prolonged the survival of mice bearing orthotopic and subcutaneous HCC tumors [52].

Tumor-associated macrophages (TAMs) play a crucial role in suppressing the anti-tumor activity of T cells or other immune-stimulating cells. Numerous studies have indicated that TAMs are typical pro-tumor macrophages responsible for the release of immunosuppressive cytokines [151]. Generally, macrophages can polarize into either the classically activated (pro-inflammatory) M1 state or the alternatively activated (anti-inflammatory) M2 state [152]. Polysaccharides from Radix Codonopsis Pilosulae (PHP-1) and Ganoderma lucidum polysaccharides (GLPS) can trigger the NF-κB and MAPK signaling pathways in the HCC microenvironment, shifting the M2 phenotype towards the M1 phenotype and alleviating immune suppression [31,32]. Research has found that Cinobufacini injection inhibits cholesterol metabolism via the AMPK/SREBP1/FASN pathway, affecting macrophage polarization, weakening hepatocellular carcinoma growth and migration, and promoting apoptosis [153].

Among the seven classes of compounds summarized in this article, natural polysaccharides have been extensively studied for their immunomodulatory effects in improving liver cancer progression, highlighting the significant potential of polysaccharides in immune regulation. Future research should explore the relationship between other types of compounds and their roles in anti-tumor activity and immune modulation, providing robust evidence for the use of immunotherapy in cancer treatment.

### 4.10. Regulates Intestinal Function

The bidirectional communication between the intestine and liver via the portal vein, bile ducts, and systemic circulation forms the gut–liver axis, a pivotal component in HCC pathogenesis. Dysfunction of intestinal barrier function and dysbiosis of gut microbiota are significant contributors to HCC development. In recent years, natural products have gained increasing attention in cancer therapy owing to their potent biological activity and minimal side effects. Leveraging the concept of the gut–liver axis, active ingredients in natural products play a crucial role in preventing and treating HCC by effectively intercepting intestinal–liver signaling pathways [154].

NPs inhibit the growth of HCC by modulating intestinal barrier function. In vivo studies have shown that Ganoderma lucidum polysaccharides can stimulate immune modulation and anti-tumor effects by regulating intestinal mucosal immune function [155]. In interventions with HCC mice established by HepG2 cells, ginsenoside Rg3 inhibits the overproduction of inflammatory factors and modulates the pathway of phosphoinositide 3-kinase, suppressing the growth of liver cancer cells [76,156]. This suggests that ginseng can inhibit the growth of HCC by suppressing inflammatory factors, increasing tight junction protein contacts, and repairing the intestinal barrier.

NPs impede the growth of HCC by enhancing gut microbiota. Recent findings have revealed a close association between gut microbiota and the occurrence, progression, and treatment of HCC, with microbial communities even discernible in late-stage tumor tissues [157]. The gut microbiota consists of probiotics and opportunistic pathogens, which promote T cell differentiation, NK cell activation, and dendritic cell maturation, actively regulating the immune system, surveilling for mutated cells, and thwarting tumor formation [158]. Studies have shown that intervention with stigmasterol in H22 tumor-bearing mice can alter the α and β diversity of the gut microbiota, significantly increasing the abundance of Lactobacillus johnsonii, Lactobacillus gasseri, and Lactobacillus vaginalis, leading to a decrease in the ratio of regulatory T cells (Tregs) to CD8+ T cells in both intestinal and tumor tissues. This enhances the immune response in the host tumor microenvironment (TME), exerting anti-tumor effects [159]. Ulva lactuca polysaccharide (ULP) inhibits tumor growth in H22 tumor-bearing mice by modulating the composition and metabolites of the gut microbiota [34]. Additionally, the Grifola frondosa polysaccharide-protein complex extracted from maitake mushrooms altered the gut microbiota composition and abundance in H22 tumor-bearing mice. This complex enriched norank_f__Muribaculaceae, Bacillus, and Bacteroides while reducing the abundance of Lactobacillus, thereby exerting anti HCC effects through gut microbiota modulation [160].

Although the interactions between the intestine and liver are not yet fully understood, intervention with NPs in liver cancer presents several advantages, including multi-target effects, comprehensive actions, multiple components, and minimal adverse reactions. However, the pathogenesis of liver cancer is exceedingly complex, requiring further elucidation of its progression and its correlation with intestinal barrier function and the gut microbiota. Moreover, the intricate nature of natural compound constituents and the diversity of therapeutic targets suggest that the specific mechanisms by which natural compounds regulate intestinal dysfunction to achieve preventive and therapeutic effects against liver cancer are still under exploration.

### 4.11. Regulation of Key Signaling Pathways

#### 4.11.1. Wnt/β-Catenin Signaling Pathway

The Wnt/β-catenin signaling pathway regulates the occurrence and development of HCC through mechanisms such as the expression of cancer-related genes, activation of hepatic stellate cells, modulation of hepatic stem cell behavior, and promotion of HCC cell invasion and metastasis [161].

Both in vivo and in vitro studies have shown that the flavonoid compound apigenin can inhibit tumor growth in HCC by modulating the Wnt/β-catenin signaling axis mediated by the long non-coding RNA H19 [56]. DEPDC1, an upregulated novel tumor antigen in HCC, is considered a molecular target for novel therapeutic drugs. Research has found that Artemisia argyi essential oil (AAEO) effectively inhibits HCC metastasis by suppressing DEPDC1 expression, weakening Wnt/β-catenin signal transduction, and inhibiting epithelial–mesenchymal transition (EMT) [118].

#### 4.11.2. PI3K/Akt Signaling Pathway

Research indicates abnormal regulation of the PI3K/Akt signaling pathway in HCC. This pathway is widely present in various biological cells and participates in regulating processes such as cell growth, survival, migration, tumor formation, and angiogenesis, all of which are closely associated with the growth, apoptosis, metastasis, and invasion of liver cancer cells [162]. Isoliquiritigenin (ISL), a flavonoid compound, induces both cell apoptosis and autophagy by upregulating the expression levels of LC3-II and cleaved-caspase-3, thereby inhibiting the PI3K/Akt/mTOR pathway [61]. Veratramine, an alkaloid, induces autophagic cell death by inhibiting the PI3K/Akt/mTOR signaling pathway, effectively suppressing the growth of HepG2 liver cancer cells both in vitro and in vivo [96]. Dandelion polysaccharides notably downregulate the protein levels of *p*-PI3K, *p*-Akt, and *p*-mTOR in HCC cells, consequently hindering HCC cell proliferation and inducing cell apoptosis through the suppression of the PI3K/Akt/mTOR pathway [45].

#### 4.11.3. JAK2/STAT3 Signaling Pathway

The transcription factor STAT3, which stands for Signal Transducer and Activator of Transcription 3, undergoes transient activation in normal cells but exhibits heightened activity in many tumor cells and adjacent cancer tissues [163]. Aberrant activation of the JAK2/STAT3 pathway is a common mechanism leading to the occurrence of liver cancer, where overexpression of STAT3 is often observed [164]. STAT3, acting as a driving factor, plays a critical role in the initiation, progression, metastasis, and immune evasion of liver cancer and is associated with poor prognosis.

The quinone compound dihydroartemisinin inhibits the activation of the JAK2/STAT3 pathway, effectively suppressing cell proliferation and promoting apoptosis, thereby inhibiting the progression of HCC [85]. The bufadienolide compound bufothionine abundant in Huachansu injection induces autophagy by upregulating the expression of autophagy-related proteins Atg5, Atg7, Beclin1, and LC3II. This is primarily achieved by inhibiting the IL-6/JAK/STAT3 pathway [101].

#### 4.11.4. Hippo–YAP Signaling Pathway

The Hippo–YAP-associated protein (YAP) signaling pathway is a recently identified pathway that holds significant importance in regulating HCC formation. It plays a critical role in controlling malignant behaviors like proliferation, apoptosis, invasion, and metastasis of liver cancer cells. Studies suggest that the alkaloid evodiamine inhibits proliferation and induces apoptosis of hepatocellular carcinoma cells through modulation of the Hippo–YAP signaling pathway [165].

Furthermore, cordycepin extracted from Cordyceps militaris promotes the expression of MST1 and LAST1 in HepG-2 cells, thereby inhibiting the expression of YAP1. Cordycepin regulates the expression of GBP3 and ETV5 by modulating the Hippo signaling pathway, thereby suppressing the proliferation and migration of HepG-2 liver cancer cells. Additionally, cordycepin regulates the expression of Bax and Bcl-2 through the Hippo signaling pathway, activating the mitochondrial apoptosis pathway. After cytochrome c is released into the cytoplasm, it activates Caspase 3, ultimately leading to cell apoptosis [166].

#### 4.11.5. NF-κB Signaling Pathway

The Nuclear Factor-κB (NF-κB) signaling pathway, a crucial transcription regulatory factor, exerts its biological effects by modulating the transcriptional expression of various genes. NF-κB plays a significant role in regulating the growth and proliferation of liver cancer cells and can contribute to spontaneous liver damage, fibrosis, and the development of liver cancer [167]. Studies have demonstrated that intervention with Cardamonin (CADMN) in xenografted nude mice results in the downregulation of proteins such as PCNA, Ki-67, and Bcl-2, alongside upregulation of the Bax protein. Furthermore, NF-κB-p65 and Ikkβ proteins are also downregulated. Thus, CADMN exerts anti-tumor effects on human liver cancer xenografts in nude mice by inhibiting the NF-κB pathway [65].

#### 4.11.6. Hedgehog Signaling Pathway

The Hedgehog signaling pathway, known for its high conservation, plays a crucial role in regulating fundamental processes like cell proliferation, differentiation, migration, and adhesion [168]. Moreover, it has been closely linked with the development of liver cancer [169]. Cepharanthine (CH), a biscoclaurine alkaloid derived from the roots of Stephania cephalantha Hayata, exhibits promising anti-tumor activity against various cancer types. Both in vivo and in vitro studies have highlighted CH’s ability to inhibit the Hedgehog/Gli1 signaling pathway by suppressing Gli1 transcription and its transcriptional activity. Additionally, CH inhibits Wnt/β-catenin signal transduction, an upstream regulatory factor of Hedgehog signaling in cancer cells treated with CH [95].

## 5. Summary and Outlook

The biological activity of NPs has ignited significant interest among researchers across pharmaceuticals, health foods, and cosmetics industries [170]. NPs, prized for their minimal side effects, high safety profile, and potent efficacy against liver cancer, are emerging as promising sources for preventive and therapeutic interventions in liver cancer.

This paper outlines seven categories of natural compounds, each demonstrating therapeutic and ameliorative effects on liver cancer. They operate through diverse mechanisms, including angiogenesis inhibition, migration and invasion suppression, cell cycle regulation, apoptosis induction, autophagy induction, ferroptosis induction, modulation of tumor metabolism, immunity regulation, intestinal function regulation, and modulation of key signaling pathways.

While considerable progress has been achieved in researching the therapeutic effects of NPs on liver cancer, several unresolved issues still demand attention.

Primarily, there is a need to augment in vivo activity experiments utilizing animal models closely resembling clinical scenarios. Concurrently, combined in vivo and in vitro studies should be conducted to delve deeply into the anticancer effects and mechanisms of action of NPs in anti-liver cancer drugs.

Furthermore, there is a call for further elucidation of the active components and structures of certain NPs. For instance, understanding the composition of natural polysaccharide monosaccharides necessitates additional steps such as purification, characterization, and modification to enhance their activity. It is imperative to systematically evaluate the toxicity and safety profiles of natural products, including supplementary studies on toxicity and relevant dosages, thus laying a robust foundation for clinical validation.

Additionally, the treatment of liver cancer necessitates targeting multiple pathways to establish a comprehensive therapeutic strategy. Some recently identified therapeutic targets are yet to be validated and translated into clinical practice. For instance, research on the tumor immune microenvironment and immunotherapy for liver cancer remains in need of extensive preclinical studies. In forthcoming research endeavors, it is not only important to explore novel treatment approaches but also to investigate the synergistic effects of combining multiple therapeutic modalities, thereby offering new avenues for the development of anti-liver cancer agents. Sustaining the quest for highly effective and low side-effect natural products for liver cancer treatment holds paramount importance. Combining different NPs might amplify the therapeutic effects against liver cancer, underscoring the significance of researching combination therapy involving NPs.

Conventional oral NPs face challenges such as low bioavailability, rapid metabolism, poor absorption, and low solubility. However, nanoparticles offer a solution by substantially enhancing the bioavailability of NPs during drug delivery, mitigating toxicity, and targeting tumor sites effectively. The nanoparticles commonly loaded with natural compounds for targeting liver cancer can be classified into organic and inorganic nanoparticles. Organic nanoparticles, primarily composed of liposomes, polymers, and polymer micelles, offer significant advantages in terms of biocompatibility, stability, and resistance to degradation by digestive enzymes. They are extensively utilized in the prevention and treatment of liver cancer [171]. For instance, the terpenoid Triptolide (TPL) presents limitations in liver cancer therapy due to its severe systemic toxicity and poor water solubility. Researchers addressed these challenges by preparing TPL-loaded membrane protein-embedded liposomes through thin-layer evaporation. Injecting these liposomes significantly inhibited hepatocellular carcinoma growth in mice with minimal damage observed in vital organs such as the liver, kidney, and others [172]. Curcumin (Cur), a well-known anticancer polyphenolic compound, faces challenges due to its poor water solubility and low bioavailability [173]. To overcome these limitations, researchers utilized d-alpha-tocopheryl polyethylene glycol 1000 succinate (TPGS) as an emulsifier to prepare Cur-loaded poly(lactic-co-glycolic acid) (PLGA) nanoparticles. This approach notably increased the concentration of Cur at the liver cancer site in mice, resulting in a reduction in tumor volume [174]. Cantharidin (CTD) has long been used in liver cancer prevention and treatment, but its direct administration entails high toxicity, and its standalone usage suffers from low bioavailability [175]. Researchers found that delivering CTD using methoxy polyethylene glycol-polylactic acid (mPEG-PLGA) micelles significantly enhances CTD’s pro-apoptotic effect on HepG2 cells. Injection of mPEG-PLGA-CTD in liver cancer mice prolongs CTD’s half-life in vivo, leading to increased accumulation at the liver cancer site [176].

Inorganic nanoparticles, primarily composed of metals and metalloids such as gold (Au) and silicon (Si), exhibit unique physical and chemical properties. Inorganic nanoparticles containing anti-tumor bioactive components show promising potential in the diagnosis and treatment of liver cancer [171]. The Chinese herbal medicine Marsdenia tenacissima (MT) possesses anticancer and hepatoprotective properties, with MT extracts demonstrating anti-hepatocellular carcinoma (HCC) activity [177,178,179]. However, due to the lack of specific targeting capability, MT extracts suffer from inadequate concentrations at tumor sites. To overcome this limitation, researchers have developed gold nanoparticles loaded with MT extracts, capable of inducing apoptosis in HepG2 cells. Furthermore, MT-gold nanoparticles exhibit superior targeting and inhibitory effects on HepG2 cells compared to MT extracts alone [180]. Due to poor water solubility and high toxicity, the clinical application of the alkaloid Hydroxycamptothecin (HCPT) is limited. Researchers have employed carboxymethyl chitosan (CMC) and hyaluronic acid (HA) modified graphene oxide (GO) as nano-carrier materials to deliver HCPT. This carrier induces cell apoptosis in vitro and demonstrates superior therapeutic efficacy against liver cancer in vivo compared to HCPT alone, suggesting its potential for targeted drug delivery in liver cancer treatment [181].

In conclusion, we aim for this review to offer a systematic and reliable summary, aiding researchers in comprehending the treatment and ameliorative effects of NPs on liver cancer. This synthesis aims to furnish a theoretical foundation for the development and application of NPs in pharmaceuticals and functional foods.

## Figures and Tables

**Table 1:** Therapeutic effects and mechanisms of natural polysaccharides on liver cancer.

No.	Name	Origin	In Vitro (a) or In Vivo (b)	Optimal Doses (/kg Body Weight)	Model	Potential Mechanism	References
1	Wild Cordyceps polysaccharides	*Cordyceps sinensis*	b	100 mg	H22 tumor-bearing BALB/c mice	Modulates immunity. Modulation of IL-10/STAT3/Bcl2 and Cytoc/Caspase8/3 signaling pathways promotes apoptosis.	[33]
2	Low molecular weight polysaccharide from Grifola frondosa	*Grifola frondosa*	b	200 mg	H22 tumor-bearing BALB/c mice	Modulates immune activity; promotes tumor cell apoptosis via the mitochondrial apoptotic pathway.	[37]
3	G. frondosa polysaccharide	*G. frondosa*	b	200 mg	H22 tumor-bearing mice	Enhances immunity and induces cell cycle arrest at G0/G1 phase.	[37]
4	Pleurotus citrinopileatus polysaccharides	*Pleurotus citrinopileatus*	b	300 mg	H22 tumor-bearing mice	Enhances immunity and induces cell cycle arrest in S phase.	[38]
5	Ganoderma lucidum polysaccharide	*Ganoderma lucidum*	a + b	200 mg	Hepatic carcinoma Hepa1-6 allograft mice; RAW 264.7 and Hepa1-6 co-culture	Regulates macrophage polarization through activating MAPK/NF-κB signaling.	[39]
6	Bletilla striata polysaccharides	*Bletilla striata*	b	200 mg	H22 tumor-bearing BALB/c mice	Modulates immunity and induces cell cycle arrest at G1 phase.	[40]
7	Acetylaminoglucan	/	b	50 mg	H22 tumor-bearing BALB/c mice	Modulates immunity and induces cell cycle arrest in S phase.	[41]
8	Bupleurum chinense DC root polysaccharides	*Bupleurum chinense* DC root	b	300 mg	H22 tumor-bearing Kunming mice	Induces S-phase block of the cell cycle and activates the mitochondrial pathway to induce apoptosis.	[42]
9	Angelica dahurica polysaccharide	*Angelica dahurica*	b	300 mg	H22 tumor-bearing BALB/c mice	Induces apoptosis by cell cycle arrest in G0/G1 phase and reduction of cellular mitochondrial membrane potential.	[43]
10	Rhodiola rosea L. root polysaccharide	*Rhodiola rosea* L. root	b	300 mg	H22 tumor-bearing mice	Induces cell cycle S-phase block by disrupting mitochondrial membrane potential and inducing apoptosis in tumor cells.	[44]
11	Dandelion polysaccharides	*Dandelion*	a + b	400 mg	H22 tumor-bearing BALB/c mice; HepG2, Huh7, and Hepa1-6 cells	Down-regulates PI3K/AKT/mTOR pathway, inhibits cell proliferation and apoptosis, and enhances immune response.	[45]
12	Dandelion polysaccharide	*Dandelion*	a + b	200 mg	H22 tumor-bearing BALB/c mice; HepG2, Huh7, Hepa1-6, and H22 cells	Inhibits IL-6-activated JAK-STAT signaling pathway; reduces hepcidin.	[46]
13	Eucommia folium polysaccharide	*Eucommia folium*	b	300 mg	H22 tumor-bearing Kunming mice	Induces S-phase block of the cell cycle and apoptosis via the mitochondrial pathway.	[47]
14	Pseudostellaria heterophylla polysaccharide	*Pseudostellaria heterophylla*	a + b	50 mg	H22 tumor-bearing C57BL/6 mice; RAW264.7 and Huh-7 cells	Regulates macrophage polarization through activating MAPK/NF-κB signaling.	[31]
15	Black fungus polysaccharide	*Black fungus*	a + b	5 mg	HCCLM3 tumor-bearing BALB/c mice; HepG2, HCCLM3, and SK-Hep1 cells	Suppresses tumor cell proliferation by promoting DNA damage, attenuating DNA damage repair, and inhibiting DNA synthesis.	[35]
16	Fucoidan	*Brown algae*	a + b	15 mg	MHCI297H tumor-bearing BALB/c mice; MHCC-97H and Hep3B cells	Causes lncRNA Linc00261 overexpression.	[48]
17	Ulva lactuca polysaccharide	*Ulva lactuca*	a + b	300 mg	H22 liver cancer tumor-bearing mice; HepG2 and H22 cells	Inhibits tumor growth through modulation of gut microbial community and metabolism and modulation of miR-98-5p/ROS signaling pathway.	[34]
18	Sipunculus nudus polysaccharide	*Sipunculus nudus*	b	200 mg	HepG2 tumor-bearing athymic nu/nu mice	Enhances immunity and induces apoptosis in tumor cells via the mitochondrial apoptotic pathway.	[49]

MAPK, mitogen-activated protein kinase; NF-κB, nuclear factor kappa-B; PI3K, phosphatidylinositide 3-kinase; AKT, protein kinase B; mTOR, mammalian target of rapamycin; IL-6, interleukin 6; DNA, deoxyribonucleic acid; lncRNA, long non-coding RNAs.

**Table 2:** Therapeutic effects and mechanisms of flavonoids on liver cancer.

No.	Name	Origin	In Vitro (a) or In Vivo (b)	Optimal Doses (/kg Body Weight)	Model	Potential Mechanism	References
1	(R)-7,3′-dihydroxy-4′-methoxy-8-methylflavane	*Resina Draconis*	a + b	20 mg	HepG2 tumor-bearing BALB/c mice and H22 tumor-bearing Kunming mice; HepG2 and SK-HEP-1 cells	Induction of apoptosis and G2/M phase block by DNA damage-driven upregulation of p21 expression in human hepatocellular carcinoma cells.	[50]
2	Neobavaisoflavone	*Psoralea*	a + b	30 mg	HCCLM3 tumor-bearing BALB/c mice; HepG2 and HCCLM3 cells	Induction of cellular pyroptosis via the tom20/bax/caspase3/GSDME pathway.	[54]
3	Isorhamnetin	/	b	100 mg	DEN + CCl4-induced HCC mice	Suppression of inflammation; regulates Akt and MAPKs to inhibit Nrf2 signaling; activates PPAR-γ and autophagy.	[55]
4	Sinensetin	citrus fruits.	b	40 mg	HepG2/C3A tumor-bearing BALB/c nude mice	Inhibition of angiogenesis in hepatocellular carcinoma by regulating VEGF/VEGFR2/AKT signaling.	[51]
5	Icaritin	plants of the Epimedium genus	b	70 mg	H22 tumor-bearing BALB/c mice	Prevention of MDSC generation via the attenuation of EMH.	[52]
6	Apigenin	parsley and chamomile	a + b	50 mg	Huh7 tumor-bearing BALB/c mice; Hep3B cells	Downregulation of H19 induces inactivation of the Wnt/β-catenin signaling pathway.	[56]
7	Lysionotin	*Lysionotus pauciflorus* Maxim	a + b	20 mg	HepG2 nude mice; HepG2 and SMMC-7721 cells	Induction of apoptosis in hepatocellular carcinoma cells via caspase-3-mediated mitochondrial pathway.	[57]
8	Kaempferide	*Mountain apple* root	a + b	25 mg	N1S1 orthotopically injected SD rats; HepG2, Huh7, and N1S1 cells	Induction of apoptosis.	[58]
9	Wogonin	*Scutellaria baicalensis*	a + b	50 mg	Orthotopically HCC-implantation mice; HepG2 cells	Inhibition of cell cycle progression by activating the glycogen synthase kinase-3 beta.	[59]
10	Total flavonoids	*Oldenlandia diffusa*	a + b	0.4 mg	H22 tumor-bearing BALB/c mice; HepG2, Hep3B, and HCCLM3 H22 cells	Induction of apoptosis and autophagy of HCC cells by inducing endoplasmic reticulum (ER) stress response and activating PERK-eIF2α-ATF4 signaling pathway.	[60]
11	Isoliquiritigenin	roots of plants belonging to licorice	a + b	50 mg	SMMC7721 tumor-bearing BALB/c mice; MHCC97-H and SMMC7721 cells	Downregulation of PI3K/AKT/mTOR pathway induces apoptosis and autophagy in hepatocellular carcinoma cells.	[61]
12	Prunetin	/	b	100 μM	DEN-induced HCC mice	Regulation of the NF-κB/PI3K/AKT signaling pathway.	[62]
13	Baicalein and baicalin	*Scutellaria* *baicalensis* Georgi	a + b	50 mg 80 mg	H22 tumor-bearing BALB/c mice; SMMC-7721 and HepG2 cells	Promotion of anti-tumor immunity by inhibiting PD-L1 expression.	[63]
14	Hesperidin	Citrus	b	200 mg	DEN-induced HCC mice	Downregulation of the PI3K/Akt signaling pathway.	[64]
15	Cardamonin	cardamom	b	50 mg	HepG2 tumor-bearing nude mice	Inhibition of NF-κB signaling pathway.	[65]
16	Safflower yellow	*Carthamus tinctorius*	a + b	5 mg	DEN-induced HCC mice; Hepa1-6 cells	Inhibition of inflammatory response; promotes collagen degradation and modulates gut microbiota to improve immune microenvironment.	[66]
17	Daidzin	soybean	a + b	100 mg	Hep3B tumor-bearing nude mice; HCCLM3 and Hep3B cells	Interference with hepatocellular carcinoma survival and migration via TPI1 and gluconeogenic pathways.	[67]
18	Salvigenin	*Scutellariae barbatae* Herba	a + b	10 μg	Huh7 tumor-bearing BALB/c mice; Human HCC cell lines	Blocking of aerobic glycolysis in HCC cells by inhibiting the PI3K/AKT/GSK-3β pathway.	[68]

DNA, deoxyribonucleic acid; DEN, diethylnitrosamine; CCL4, carbon tetrachloride, HCC, hepatocellular carcinoma, Akt, protein kinase B; MAPK, mitogen activated protein kinase, VEGF, vascular endothelial growth factor; MDSC, myeloid-derived suppressor cells; EMH, extramedullary hematopoiesis; H19, long non-coding RNAs H19; SD, sprague dawley; PI3K, phosphatidylinositide 3-kinase; NF-κB, nuclear factor kappa-B; “/” indicates that the reference is not mentioned or is unclear.

**Table 3:** Therapeutic effects of terpenoids on liver cancer and their potential mechanisms.

No.	Name	Origin	In Vitro (a) or In Vivo (b)	Optimal Doses (/kg Body Weight)	Model	Potential Mechanism	References
1	Ginsenoside Rh4	ginseng	a + b	/	HCC tumor-bearing BALB/c mice; HUH7 and LM3 cells	Inhibition of inflammation-associated HCC progression by targeting HDAC4/IL-6/STAT3 signaling.	[74]
2	Octyl ester derivative of ginsenoside Rh2 (Rh2-O)	ginseng	b	10 mg	H22 tumor-bearing Tlr4^−/−^ mice	Anti-hepatocellular carcinoma through TLR4-mediated immunomodulation of lymphocytes.	[78]
3	Ginsenoside Compound K	ginseng	a + b	5 mg	DEN-induced SD mice; Bel-7404 and Huh7 cells	Regulation of HIF-1α-mediated glycolysis by Bclaf1 inhibits cell proliferation.	[75]
4	Ginsenoside Rk3	ginseng	a + b	100 mg	HCC-LM3 tumor-bearing BALB/c mice; HepG2 and HCC-LM3 cells	Inhibition of cell proliferation and induction of cell cycle arrest. Promotion of cell autophagy and apoptosis via PI3K/AKT.	[76]
5	Ginsenoside Rh2	ginseng	a + b	30 μmol	H22 tumor-bearing C57BL/6 mice; Huh-7 and H22 cells	Suppression of hepatocellular carcinoma invasion and metastasis by inhibition of c-Jun/COX2/PGE2 pathway-mediated EMT.	[77]
6	Ginsenoside CK	ginseng	a + b	60 mg	HCC-LM3 tumor-bearing BALB/c mice; HepG2, SMMC-7721, HCC-LM3, and Huh-7 cells	Inhibition of hypoxia-induced epithelial mesenchymal transition in hepatocellular carcinoma through the HIF-1α/NF-κB feedback pathway.	[81]
7	Saikosaponin-b2 (SS-b2)	*Radix bupleuri*	a + b	20 mg	H22 tumor-bearing C57BL/6 mice; HepG2 and HUVECs cells	Inhibition of angiogenesis through inhibition of the VEGF/ERK/HIF-1α signaling pathway.	[82]
8	Saikosaponin-b2 (SS-b2)	*Radix bupleuri*	a + b	6 mg	DEN-induced BALB/c mice; RAW 264.7 macrophages	Upregulation of STK4 inhibits IRAK1/NF-κB signaling axis effectively suppresses PLCs.	[83]
9	Astragaloside IV (AS-IV)	*Astragalus membranaceus*	a + b	40 mg; 20 μmol/L	HT, genotype: pSmad3C^+/−^; HO, genotype: Nrf2^−/−^; lentivirus-transfected HepG2 cells	Amelioration of hepatocellular carcinoma by Nrf2-mediated transformation of pSmad3C/3L.	[84]
10	Dihydrotanshinon	*Salvia miltiorrhiza Bunge*	a + b	15 mg; 4 μg/mL	SMMC7721 tumor-bearing Balb/c mice; HCCLM3, SMMC7721, Hep3B. and HepG2 cells	Promotion of apoptosis by blocking the JAK2/STAT3 pathway.	[85]
11	Betulinic acid	bark of several plants	a + b	40 mg	HUH7 tumor-bearing BALB/c mice; HUH7 and HCCLM3 cells	Inhibition of hepatocellular carcinoma cell growth through activation of NCOA4-mediated ferritin phagocytosis pathway.	[79]
12	2α, 3α, 23-trihydroxy-urs-12-en-28-oic acid,	*Ganoderma lucidum*	b	400 mg	S180 and H22 tumor-bearing Kunming mice	Amelioration of lipid peroxidation. Down-regulation of Bcl-2 and up-regulation of Bax. Increase in intestinal flora richness and structure.	[80]
13	Pseudolaric acid B	*Pseudolarix kaempferi*	a + b	20 mg	Hepa1–6 tumor-bearing C57BL/6 mice; Hepa1–6 cells	Triggering apoptosis through activation of the AMPK/JNK/dr P1/mitochondrial fission pathway.	[86]
14	Mallotucin D	*Croton crassifolius*	a	20 μM	HepG2 cells	Induction of cellular pyroptosis. Inhibition of the PI3K/AKT/mTOR pathway activates mitochondrial autophagy.	[87]
15	Heteronemin	*Hippospongia* sp.	a	20 μM	HA22T and HA59T cells	Induction of apoptosis via the caspase pathway. Induction of ferroptosis by down-regulation of GPX4.	[88]

HCC, hepatocellular carcinoma; HDAC4, histone deacetylase 4; IL-6, interleukin 6; STAT3, signal transducer and activator of transcription 3; DEN, diethylnitrosamine; SD, sprague-dawley; PI3K, phosphatidylinositide 3-kinase; AKT, protein kinase B; PGF2, prostaglandin F2; EMT, epithelial-mesenchymal transition; HIF-1, hypoxia inducible factor-1; NF-κB, nuclear factor kappa-B; VEGF, vascular endothelial growth factor; ERK, extracellular regulated protein kinases; PLC: primary liver cancer; HT genotype, pSmad3C^+/−^: Smad3 *C*-terminal phosphorylation site-heterozygous mutant mice model; HO genotype, Nrf2^−/−^: Nrf2 conventional knockout homozygous mice; JAK2, janus kinase-2; Bcl-2, B-cell lymphoma-2; AMPK, AMP-activated protein kinase; JNK, c-Jun N-terminal kinase; GPX4, glutathione peroxidase 4; “/” indicates that the reference is not mentioned or is unclear.

**Table 4:** Therapeutic effects and potential mechanisms of alkaloids on liver cancer.

No.	Name	Origin	In Vitro (a) or In Vivo (b)	Optimal Dose (/kg Body Weight) or Concentration	Model	Potential Mechanism	References
1	Matrine	*Sophora flavescens* Aition	b	100 mg/kg matrine and 2 mg/kg cisplatin.	HepG2 tumor-bearing BALB/c mice	Promotion of apoptosis via suppression of survivin and activation of the caspase pathway.	[94]
2	Cepharanthine	*Stephania cephalantha* Hayata	a + b	20 mg; 60 µM	Huh7 tumor-bearing BALB/c mice; Huh7 and HepG2 cells	Inhibition of the Wnt/β-catenin/Hedgehog signaling pathway.	[95]
3	Veratramine	*Veratrum nigrum* L.	a + b	2 mg; 10, 20 and 40 µM	HepG2 tumor-bearing BALB/c mice; HepG2 cells	Activation of autophagy-mediated apoptosis through inhibition of the PI3K/Akt/mTOR signaling pathway.	[96]
4	Anisodamine	*Anisodus*	b	200 mg	HepG2 tumor-bearing BALB/c mice	Inhibition of NLRP3 inflammatory vesicles; induction of apoptosis.	[91]
5	Chaetocochin J	*Chaetomium* sp.	a + b	0.5 mg	HepG2 tumor-bearing nude mice; HepG2 and Hep3B cells	Inhibition of the PI3K/Akt/mTOR/p70S6K/4EBP1 pathway. Disruption of HIF-1α/p300 binding.	[97]
6	Sophoridine	*Sophora* *alopecuroides* L.	a + b	50 mg	HepG2 LR tumor-bearing BALB/c mice; HepG2 and Huh7 cells	Inhibition of the RAS/MEK/ERK axis by decreasing VEGFR2 expression.	[98]
7	Stachydrine hydrochloride	*Panzeria alaschanica* Kupr	a + b	30 mg	Patient-derived xenograft model; SMMC-7721 and HepG2 cells	Regulation of LIF/AMPK induced autophagy and senescence.	[92]
8	Cyclovirobuxine D	*Buxus microphylla*	a + b	10 mg	HepG2 tumor-bearing BALB/c mice; HepG2 and HCCLM3 cells	Suppression of cell proliferation, migration, and invasion through inhibition of the EGFR-FAK-AKT/ERK1/2-Slug signaling pathway.	[99]
9	Piperlongumine	*Piper longum* L.	a + b	10 mg/kg PL and 5 mg/kg sorafenib	HCCLM3 tumor-bearing BALB/c mice; HCCLM3 and SMMC7721 cells	Mediation of ROS-AMPK activation and targeting of CPSF7.	[100]
10	Bufothionine	cinobufacini injection	a + b	343.35 μg/kg	H22-tumor-bearing Kunming mice; SMMC7721 cells	Induction of autophagy in HCC by inhibiting JAK2/STAT3 pathway.	[101]
11	Abrine	*Abrus cantonments*	a + b	15 mg	HepG2 tumor-bearing BALB/c mice; HepG2 and Huh7 cells	Regulation of hepatocellular carcinoma cell growth and apoptosis through the KAT5/PD-L1 axis and regulation of T cell proliferation and activation.	[102]
12	Cepharanthine	*Stephania cepharantha* Hayata	a + b	20 mg	Hep3B tumor-bearing nude mice; Hep3B and HCCLM3 cells	Inhibition of HCC cell proliferation by regulating amino acid metabolism and cholesterol metabolism; promotion of apoptosis and necrosis.	[93]
13	Ventilagolin	*Ventilago leiocarpa* Benth	b	12 mg	SMMC-7721 tumor-bearing BALB/c-nu nude mice	Inhibition of HCC cell growth, migration, and invasion by regulating Pim-1 expression and EMT markers.	[103]

PI3K, phosphatidylinositide 3-kinase; AKT, protein kinase B; mTOR, mammalian target of rapamycin; NLRP3, NOD-like receptor thermal protein domain associated protein 3; RAS, renin-angiotensin system; MEK, mitogen-activated extracellular signal-regulated kinase; ERK, extracellular regulated protein kinases; LIF, leukemia inhibitory factor; AMPK, AMP-activated protein kinase; EGFR, epidermal growth factor receptor; FAK, focal adhesion kinase; ROS, reactive oxygen species; HCC, hepatocellular carcinoma; JAK2, just another kinase2; STAT3, signal transducer and activator of transcription 3; PD-L1, programmed cell death 1 ligand 1; EMT, epithelial–mesenchymal transition.

**Table 5:** Therapeutic effects and potential mechanisms of polyphenols on liver cancer.

No.	Name	Origin	In Vitro (a) or In Vivo (b)	Optimal Dose (/kg Body Weight) or Concentration	Model	Potential Mechanism	References
1	Tannins	*Terminalia bellirica*	b	2 g	HepG2 tumor-bearing ICR mice	Regulation of the EGFR signaling pathway and modulation of the immunosuppressive tumor microenvironment.	[106]
2	Proanthocyanidin-B2	peanut skin	a + b	300 mg	DEN + CCl4-induced HCC mice; Huh7 and Smmc-7721 cells	Inhibition of AKT leads to cell cycle arrest and tumor cell metabolic suppression.	[111]
3	Procyanidin B1	grape seed	b	15 mg	HepG2 tumor-bearing Balb/c mice	Inhibition of tumor growth in mice by inhibiting Kv10.1 current.	[112]
4	Chlorogenic acid	*Eucommia ulmoides* Oliver	a	0, 30 and 300 µM	HepG2 and Huh-7cells	Inhibition of the NF-κB signaling pathway and triggering of mitochondrial apoptosis.	[107]
5	Curcumae	turmeric	b	10 mg	DEN-induced albino Wistar rats	Regulation of oxidative stress, inflammatory response, and gut microbiota.	[109]
6	Curcumin	turmeric	a	0, 5, 10 μM	SMMC-7721 and HepG2 cells	Down-regulation of DJ-1 inhibits cell proliferation.	[108]
7	Resveratrol		a + b	100 mg	HepG2 tumor-bearing BALB/c nude mice; HepG2 and Hep3B cells	Down-regulation of MARCH1 regulates PTEN/AKT signaling.	[110]

DEN, diethylnitrosamine; HCC, hepatocellular carcinoma; EGFR, epidermal growth factor receptor; AKT, protein kinase B; NF-κB, nuclear factor kappa-B; MARCH, membrane associated RING-CH; PTEN, phosphatase and tensin homolog.

**Table 6:** Therapeutic effects and potential mechanisms of quinones on liver cancer.

No.	Name	Origin	In Vitro (a) or In Vivo (b)	Optimal Dose (/kg Body Weight) or Concentration	Model	Potential Mechanism	Reference
1	Emodin	*Rheum palmatum* L.	a	0, 25, 50, 75, and 100 μM	HepG2 cells	Induction of autophagy and inhibited EMT through inhibition of PI3K/AKT/mTOR and Wnt/β-catenin pathways.	[114]
2	Emodin succinyl ester	*Rheum palmatum*	b	200 mg	DEN-induced HCC mice	Inhibition of cell proliferation and migration through inhibition of AR interaction with EZH2.	[115]
3	Thymoquinone	*Nigella sativa*	b	5 mg	DEN-induced HCC mice	Inhibition of angiogenesis by targeting miR-1-3p.	[116]
4	Plumbagin	*Plumbagin zeylanica* L	b	2 mg	HepG2 tumor-bearing Balb/c mice	Inhibition of USP31 activity leads to GPX4 protein degradation and apoptosis.	[117]

EMT, epithelial–mesenchymal transition; PI3K, phosphatidylinositide 3-kinase; AKT, protein kinase B; mTOR, mammalian target of rapamycin; DEN, dimethylnitrosamine; HCC, hepatocellular carcinoma; EZH2, enhancer of zeste homolog; GPX4, glutathione peroxidase 4.

**Table 7:** Therapeutic effects and potential mechanisms of volatile oil on liver.

No.	Name	Origin	In Vitro (a) or In Vivo (b)	Optimal Dose (/kg Body Weight) or Concentration	Model	Potential Mechanism	Reference
1	Artemisia argyi essential oil	*Artemisia argyi*	a + b	230 mg	HepG2-Luc tumor-bearing BALB/c mice; HepG2 and SMMC-7721 cells	Inhibition of the Wnt/β-catenin signaling pathway suppresses metastasis of hepatocellular carcinoma.	[118]
2	D. pycnastera leaf essential oil	*Duguetia pycnastera* Sandwith	a + b	40 mg	HepG2 tumor-bearing C.B-17 SCID mice; HepG2 cell	Inhibition of tumor growth.	[119]
3	Conobea scoparioides essential oil	*Conobea scoparioides*	a + b	80 mg	HepG2 tumor-bearing C.B-17 SCID mice; HepG2 cell	Induction of apoptosis and inhibition of tumor growth.	[120]
4	Aniba parviflora essential oil	*Aniba parviflora*	a + b	80 mg	HepG2 tumor-bearing C.B-17 SCID mice	Inhibition of tumor cell proliferation and inhibition of tumor growth.	[121]
5	C. articulatus rhizome essential oil	*Cyperus articulatus* L	b	80 mg	HepG2 tumor-bearing C.B-17 SCID mice	Blocking the cell cycle and inducing apoptosis.	[122]
6	Essential oil of lemon myrtle	*Lemon myrtle*	a	40.90 µM	HepG2 cells	Blocking the cell cycle and inducing apoptosis	[123]
7	Pogostemon cablin essential oils	*Pogostemon cablin*	a + b	200 mg	HepG2 tumor-bearing Balb/c nude mice; HCC, SVEC, MDCK, and BNL CL.2 cells	Blocking the cell cycle and inducing apoptosis.	[124]

SCID, severe combined immunodeficient.

## Data Availability

Not applicable.
